# Factors Affecting Online Chinese as a Foreign Language Learning Stickiness: A Study on International Students in China

**DOI:** 10.3389/fpsyg.2021.803669

**Published:** 2022-01-20

**Authors:** Chen Chen

**Affiliations:** International College, Southwest University, Chongqing, China

**Keywords:** learning stickiness, Chinese as a foreign language, expectancy confirmation, online teaching and learning, international student

## Abstract

This study explored international students’ online Chinese as a foreign language learning stickiness in a Chinese university context. A new theoretical model was designed and verified to serve the mixed-method investigation. Participants were a group of 194 international students learning CFL online in a Chinese university. Data were collected through an online questionnaire for all the students and semi-structural individual interviews with eight volunteer participants. The structural equation modeling was conducted to analyze the collected quantitative data, and content analysis was used for the qualitative interview. Findings revealed that the online learning stickiness was significantly impacted by students’ learning expectancy confirmation, which was closely correlated with three key factors: academic integration, social integration, and technological factors. Moreover, social integration was found to be a direct contributor to learning stickiness. These results highlighted the importance of the high quality of the curriculum, the harmonious learning atmosphere, and the need for technical preparations and training for online Chinese language teaching and learning. The study also emphasized the need of integrating social interaction into Chinese learning in an online context. Investigation of a wider range of Chinese learners was recommended for future studies on learning stickiness and the new online approach.

## Introduction

Over the past two decades, Chinese as a foreign or second language (CFL/CSL) has been widely taught and learned within and outside China (Ma et al., 2017; Gong et al., 2018, 2020a,2020c). Up to 2019, more than 500,000 international students have been enrolled in 820 higher educational institutions across China (Ministry of Education China, 2018). To fulfill international students’ academic and causal needs in China, various Chinese language learning programs have been set both in-class and online. In recent years, online CFL teaching and learning has served as an important approach in addition to routine in-class one, helping many international students achieve their language development goals (Jin, 2018; Gong et al., 2020b). However, to achieve teaching and learning CFL online also faces a range of challenges, such as technical problems, learning habits and strategies, and some socioeconomic factors (Simamora, 2020). To keep students’ learning sustainable and to decrease their dropout rates is one of the key considerations for all participants, including administrators, teachers, courseware developers, and students, in teaching and learning.

A dropout in online learning refers to a student that he/she inconspicuously discontinues participation in any learning activities, and he/she will not return to learning in any way in the future (Rodríguez, 2012). Studies have noticed that for most Massive Online Open Courses (MOOCs, such as Coursera, Udacity, and EdX), students’ completion rates are only around 10% (Lushnikova et al., 2012; Harrison, 2013), meaning that the overwhelming majority of their online students dropped out. A large-scale investigation of 58 online courses also figured out 86.5% dropout rates of 188,802 registered participants (Scopeo, 2013). A similar challenge is noticed in CFL teaching and learning. Teachers have a complaint about international students’ high dropout rates, particularly in an online context (Tsui et al., 2017).

Recently, the concept of learning stickiness is employed to explore dropout issues in the online context (e.g., Chen, 2014; Liu and Pu, 2020). Learning stickiness refers to the ability of online learning to attract and hold the attention of learners (Robinson and Cook, 2018). With high learning stickiness, a student would have a compelling and magnetic reason to engage in learning activities actively and return to learning in the future autonomously (Geer and Barnes, 2006). When a student becomes sticky with the learning, he/she is usually more deeply and frequently involved in learning and practice. Compared with peers, intensive exposure to learning and practices would benefit a sticky learner’s language knowledge and skills development in return (Hsu and Liao, 2014).

For online learning, stickiness can serve as an important way to explore students’ learning willingness and engagement. Previous studies investigating students’ learning stickiness have been conducted in general education contexts (Geer and Barnes, 2006; Xu et al., 2017; Robinson and Cook, 2018). However, few studies have attempted to investigate the factors that may have an influence on CFL students’ learning stickiness in an online context. In addition, most current studies have explored students’ engagement in learning from a qualitative perspective (Wang et al., 2019). Yang et al. (2018) highlighted the importance of a combination of both quantitative and qualitative methods in the investigation of learning stickiness, particularly in a new online context where several latent factors might be intertwined. To echo the call for facilitating language development of international students in the new era, it is important to explore the factors that may encourage students to become sticky with the new online approach with new designs.

Given the above-mentioned gaps, the present study explored the learning stickiness of international students in an online teaching and learning context. It aimed at figuring out factors that had an influence on international students’ participation in online CFL learning activities. The study also examined how these factors interacted with each other, as well as their collective contributions to students’ willingness of future engagement in online learning. This hopefully helps students and teachers improve the quality of online courses, select appropriate teaching strategies, and pave the way for sustainable development of online CFL teaching and learning.

## Literature Review

### Learning Stickiness

The concept of stickiness is originally used to describe the characteristics of commercial websites, referring to the relationship between websites and users. A website with high stickiness often attracts users to visit repeatedly, to spend a long time on it, and to purchase its products and services (Zott et al., 2000). Users, in the meanwhile, are satisfied with the experience. They intend to be “loyal customers,” and usually have a greater likelihood of revisiting and reusing their sticky website. A number of empirical studies have been conducted by employing the concept of stickiness in various fields, indicating its uses to reveal the mutual relationship between the provided services and users (Lin et al., 2010; Xu and Liu, 2010). Ways to increase user stickiness were also highlighted in these studies, such as the promotion of the website quality, the quick responses of user services, and the easy access for online users. Drawing from these empirical investigations, it has been generally accepted that stickiness can be used as a key factor to effectively predict the engagement of users in online activities, as well as their willingness of future return to the platform (Lin, 2007).

Online learning shares common points with website visits: students log in, select the content, enjoy the learning, acquire some information, and return in a few days (or never return). In this respect, the concept of stickiness could be extended to the field of online learning. From a learner perspective, with stickiness, he/she usually learns online repeatedly and continuously, with more investment into learning activities before achieving his/her learning goals. From a learning perspective, to increase stickiness, online learning caters for the needs of its “loyal learners”: to modify its content in accordance with learners’ expectations, to provide user-friendly services, and to help solve potential obstacles. As Robinson and Cook (2018) have elaborated, learning stickiness means the “Velcro sort of relationship” (p. 5) between learning and learners. On this train of thought, the present study used the concept of learning stickiness to explore the mutual relationship between online learning and international students from both the learner and the learning dimensions.

Dropout has long been a concern in language learning for both teachers and students, particularly in an online context where less teacher supervision was implemented (Chen, 2021). Learning stickiness, as one of the external realizations of students’ intention of learning desire and engagement, could play a role in predicting students’ learning dropout. It hopefully helps teachers and students to locate factors leading to learning dropout and to figure out ways to increase students’ stickiness and to get them back to learning activities.

### The Theoretical Model

A wide range of studies have investigated online learning engagement, persistence, and dropout, and confirmed that a bunch of learner factors, such as learner satisfaction, expectation confirmation, and perceived usefulness of online learning, affected learning stickiness (Lien et al., 2017; Yu et al., 2017; Lü and Lü, 2020). Considering the nature of online learning as a kind of repeated game (Alyaz et al., 2017), the present study highlighted the focuses not only on the learner’s first attempt at online learning but also on repeated behaviors after acceptance.

**FIGURE 1:**
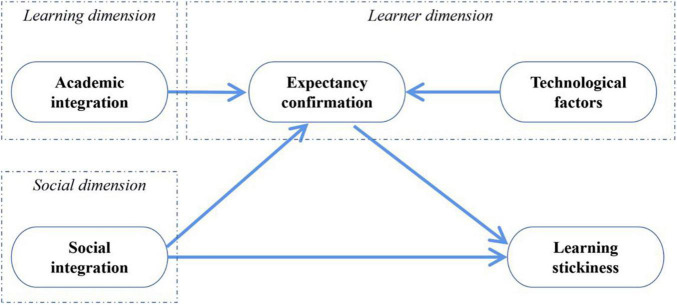
The theoretical model of present study.

It should be noticed that many previous investigations of online learning stickiness largely ignored the non-learner impacts (Halilovic and Cicic, 2013). These factors, such as learning resources, peers, teachers, devices, learning environment, etc., play an important role in online foreign language learning (Lin et al., 2021). Considering the social nature of online teaching and learning, Social Cognition Theory (SCT) can be applied into the context to serve the exploration of learner’s engagement from both learner and non-learner perspectives (Bryant et al., 2005). The theory proposes that the learning decision is made in accordance with the influence of a learner’s individual factors and his/her involved social environment. Being enlightened by SCT, this study constructs a new theoretical model and classifies the latent affecting factors of online learning stickiness into three dimensions: learning dimension, social dimension, and learner dimension. Based upon a review of current literature, four constructs were incorporated into the model, which are academic integration, social integration, technological factors, and expectancy confirmation (see Figure 1).

### Learning Stickiness and Its Affecting Factors

Attempts have been made to find factors influencing learning stickiness in various educational contexts. At the early stage, focuses were placed on traditional in-class learning. For instance, Bean and Metzner’s (1985) student attrition model and Tinto’s student integration model (1993) guided the early attempts of learning stickiness research. Several factors that might lead to low learning engagement and high dropout rates were specified in those empirical studies, including educational environment, learning content, general individual factors, and psychological stats.

However, considering the differences between the new teaching and learning approaches and the traditional ones, these models have “limited applicability” (Rovai, 2003) in an online learning context. Learning dropout of online learners with more individually different elements could be barely explained by old models. Then several new models contextualized in online teaching and learning were proposed and employed for studies on learning stickiness (Guo et al., 2010; Kim and Lee, 2017; Yu et al., 2017; Leem and Sung, 2019). These models attempted to explain and predict the issues of learning stickiness and dropout via four variables, which are individual factors (learner characteristics and skills), learning internal factors (academic integration, social integration, self-esteem, interpersonal relationship, etc.), and learning external ones (financial support, learning environment, available learning time, etc.).

In light of these theoretical models, researchers have conducted a number of empirical studies exploring the secrets of stickiness in an online learning context from various perspectives: from a learner perspective, Wu et al. (2010a) and Kim and Lee (2017) portrayed typical successful online learners and highlighted the close relationship between learner’s satisfaction and learning stickiness; from a learning perspective, Rienties et al. (2014) stressed the effects of introducing qualified academic content to keep students learning, while Lü and Lü (2020) also highlighted the importance of high quality of learning resources in decreasing dropout rate; and from an environment perspective, Willging and Johnson (2004); So and Brush (2008), and Wu et al. (2010b) listed a series of external affecting factors, including time conflict and technology literacy as key factors contributing to online learning stickiness. Others, including curriculum-related factors, like the organization of curriculum, test scores, in-time instructions, and social factors, like peer interaction and teacher supervision, were also found to be responsible for students’ stickiness in an online education context (Choi and Park, 2018).

Learning from previous studies, it can be seen that a wide range of factors may impact online learning stickiness, whereas some factors mentioned by previous studies, like learner characteristics and learner skills, might not be applied to learning due to little solid evidence of their significance (Willging and Johnson, 2004). Contextualized in a new online context, investigation of all scattered factors in one study could hardly provide a systematic picture of learning stickiness, nor supply valuable data for further mining on the topic. Therefore, this study introduced a new theoretical model to serve the exploration of learning stickiness in an online CFL teaching and learning context.

In the learning dimension, academic integration was investigated as the main construct. Academic integration includes the quality of courses, teacher instructions, the organization of learning resources, and other learning-related elements (Choi and Park, 2018). This construct focuses on whether online learning could fulfill learners’ academic needs, improve academic performances, and elevate learning efficiency. It has been considered as the most basic demand of learners in terms of using the learning platform. Studies have noticed the influence of academic integration on students’ decisions of engagement: Lü and Lü (2020) found a positive correlation between academic integration and learners’ interests in online learning; Zheng and Chen (2011) confirmed its contribution to learners’ satisfaction; Packham et al. (2004) believed that unsuccessful academic integration would compel learners to leave without returning to online learning.

It should be noted that, however, academic integration is assumed to have no direct impact on students’ stickiness of language learning. Stickiness is considered as a kind of mutual relationship between learning and students, not just the unilateral loyalty of one party to the other. “Both the student and the activity each provide the relevant surface to which to adhere; for the student to stick to the learning and the learning to ‘stick’ to the student” (Robinson and Cook, 2018, p. 5). Studies have noticed that the high quality of online learning attracts students to engage with the learning content, but it may fail to motivate students to act or to interact with others (Bhattacherjee, 2001; Garrett, 2007). That may result in the “one-side stickiness,” which is considered as the “incomplete” stickiness (Robinson and Cook, 2018). As the learning dimension focuses on the content and the quality of the learning approach, it is not directly connected with learning stickiness. It is more a concept investigating learners’ will and behavior (Waheed et al., 2016). Instead, the learning dimension has an impact on learning stickiness as the learner works as a mediating role. It was hypothesized that with high academic integration, online CFL learning increases learner’s satisfaction and recognition, but not directly increases learner’s learning stickiness.

Social integration, the interaction between a learner and his/her social environment (Vygotsky, 1978), has been widely recognized as a key facilitator in cognitive development. For foreign language learners, social integration usually creates a supportive environment by incorporating social interaction into learning, including peers, teachers, and extern supports. Studies have indicated the influence of social integration on increasing learning stickiness in an online context: Jiang et al. (2010) believed that interaction with others in an authentic social environment could positively affect online user’s trust and engagement in the platform; Wu et al. (2011) found that online interaction increased learner’s confidence, motivation, and ability, leading to their willingness to engage in future learning; Yang and Lin (2020) highlighted the importance of interaction as well. In the respect of the social dimension of online foreign language learning, social integration is considered as a key element regarding learners’ stickiness.

In the learner dimension, two major constructs were examined in the present study. Technological factors are the basic conditions for online learning. A wide range of factors regarding learning access and support, such as affordable devices for online learning, a stable Internet-connected learning environment, and learners’ technical literacy, are latent affecting factors, leading to different learning engagement, experiences, and performances (Rovai, 2003). Although these factors are not directly related to foreign language development, they may result in learners’ active or passive withdrawal from learning (Pierrakeas et al., 2004). Learning stickiness is about students’ repeated and continuous engagement in learning activities (Robinson and Cook, 2018). While technological factors may have an impact on students’ learning experiences, these factors are not directly affecting learning stickiness for students may find the best ways to access online learning by evaluating their Internet connection and hardware conditions (Chen, 2014). Otherwise, they would abandon the learning after the first or second attempts, instead of investing in learning repeatedly. Assuming their influence on learning stickiness in an online context while this impact is not direct, this study framed technological factors into the model.

Expectancy confirmation is also a factor to assess to what extent online learning could fulfill its learner’s expectations. Keengwe et al. (2012) figured out a positive connection between learner’s expectancy confirmation and satisfaction, and suggested that learning expectancy confirmation led to learner’s retention; Alshurideh et al. (2020) found that expectation confirmation affected students’ intention to engage in mobile-based learning; Wang et al. (2021) also draw a similar conclusion in an empirical investigation in China. As Craig et al. (2008) has claimed, when a learner’s expectations of online learning are high, he/she will recognize the quality of the learning content, and have active engagement in learning; and in turn, a learner decides to stay when the expectations are achieved through his/her learning with qualified resources. Expectancy confirmation plays a central and mediating role in the emergence of stickiness in students’ learning process. Besides, technology-related factors can also have an impact on students’ realization of their learning expectations, which may be an affecting factor of their final decisions of persistence in learning. Enlightened by previous studies, the present one incorporated the construct of expectancy confirmation into the framework.

Although there is an extensive body of literature in learning stickiness and factors that may have some influence on it, focuses are seldom paid to a CFL area. Learners, teachers, and educators of CFL have been worried about online learning dropout for a long time, while not many empirical studies have been conducted, with fewer theoretical models being incorporated. Considering these gaps, the present study used the new theoretical model and addressed two research questions below:

RQ1: What factors influenced online CFL learning stickiness of international students in China?

RQ2: What were the relationships among these affecting factors of online CFL learning stickiness?

## Methodology

### Research Context and Participants

A total of 200 international students enrolled in a typical public university located in southwestern China voluntarily participated in this empirical study. As a center for Chinese language and culture education, this university provides a wide range of online and offline CFL programs, courses, and resources for international students with diverse educational backgrounds and different native languages. Up to the commencement of the study, all of them had passed Hanyu Shuiping Kaoshi (HSK) Level 5, which is equivalent to Advanced-Mid to Advanced-High of American Council on the Teaching of Foreign Languages (ACTFL). Table 1 shows the demographic information of the student participants (194 valid responses).

**TABLE 1:** The demographic information of participants (*n* = 194).

Item	Classification	Number	Percentage
Age	Under 20	60	30.9%
	20–29	107	55.2%
	30–39	22	11.3%
	Over 40	5	2.6%
Gender*	Male	71	36.6%
	Female	120	61.9%
Major	Botany	16	8.2%
	Computer science	28	14.4%
	Economics and finance	71	36.6%
	Education	45	23.2%
	Engineering	11	5.7%
	Psychology	23	11.9%
L1 background	English	41	21.1%
	French	50	25.8%
	Spanish	25	12.9%
	Arabic	33	17.0%
	Thai	18	9.3%
	Others	27	13.9%
Experience of CFL learning	1–3 years	47	24.2%
	4–6 years	59	30.4%
	7–9 years	64	33.0%
	More than 10 years	24	12.4%
Experience of online learning	1–2 years	38	19.6%
	3–4 years	88	45.4%
	5–6 years	45	23.2%
	More than 7 years	23	11.9%
Online accessibility and hardware conditions	Excellent	77	39.7%
	Average	86	44.3%
	Poor	31	16.0%

*Three participants preferred not to mention their gender in the questionnaire.

To capture international students’ experience and perceptions of their engagement in and withdrawal from online CFL learning, individual interviews were employed in the present study. After their accomplishment of the questionnaire, eight volunteer students with different educational backgrounds and CFL learning experiences were selected and interviewed. The details of the interviewees can be seen in Table 2.

**TABLE 2:** The demographic information of the interviewees (*n* = 8).

Pseudonym	Age	Gender	Nation	Major	Experience of CFL learning	Experience of online learning
Geng	31	Male	Korea	Economics and finance	15 years	2 years
Qiu	21	Female	Thailand	Computer science	11 years	2 years
Bai	22	Female	Malaysia	Economics and finance	8 years	5 years
Yang	18	Female	Russia	Education	5 years	4 years
Jiang	20	Male	Egypt	Engineering	3 years	3 years
Chen	24	Male	Canada	Psychology	4 years	7 years
Liu	22	Male	Vietnam	Education	4 years	1 year
Zhang	41	Female	Chile	Botany	10 years	3 years

Online Comprehensive Chinese courses were investigated in the present study. The courses were compulsory ones for all international students in the university. Students’ all four language skills and their knowledge of the Chinese language, history, and culture were trained and improved in the courses. The courses were selected for the study could cater for international students’ overall language development, and provide a complete picture of their online language learning of different content. Student participants attended the courses for at least 6 h per week. WeChat and DingTalk were employed as the major tools for online teaching and learning. Affected by the COVID-19 pandemic, all participants had been learning CFL online for at least 1 year. They were supposed to be familiar with the online approach with abundant experiences and personal thoughts on it.

### Instruments

Researchers have claimed that a mixed-methods study that combines both the quantitative and qualitative methods is a promising direction for learning stickiness research; hence, this method deserves academic attention (Alshurideh et al., 2020). A mixed-method research design was adopted in the present study, because of its value to achieve an elaborate and comprehensive understanding of international students’ learning stickiness in an online CFL context. An online questionnaire for all the participants and semi-structural individual interviews with eight volunteer students were employed. This study adopted a mixed-methods approach. The quantitative data were collected via an online questionnaire and analyzed first, and then, the qualitative data were used to explain, clarify, illustrate, and elaborate on the quantitative findings (Dornyei, 2007; Ivankova and Creswell, 2009). The qualitative data from interviewees’ descriptions could play a role to figure out what lied behind an educational issue from a learner perspective. Evidence from different data gatherings and analysis methods enhanced the internal validity of the research, which could provide a solid basis for data triangulation in a technology-based context to minimize misunderstanding and to enhance the data validity (Ashour, 2018).

The questionnaire surveyed the student participants’ experiences and thoughts on learning stickiness and dropout in online CFL learning. Items were developed by referring to numerous prior relevant studies and modified in accordance with the theoretical model of the current study and the online CFL teaching and learning context. Items for academic integration were derived from Lin (2007) and Wu et al. (2010a). Items for social integration were mainly extracted and modified from Chou and Liu (2005). The technological factors were surveyed via items developed from Venkatesh et al. (2003) and Wu et al. (2010a). Items for learner’s expectancy confirmation were elicited and modified from Chiu et al. (2005) and Wu et al. (2010b). Items for learning stickiness were consulted (Chen, 2014) in developing the questionnaire as well.

The initial questionnaire was refined through iterative consultations with two experts in the field of educational psychology. The process enabled the researcher to gauge the clarity of the instrument and to verify that the items could convey the actual intention of the study. This process continued until no further questions were raised. Minor changes were made to the initial version. The revised questionnaire was employed in the formal investigation of the present study. The final questionnaire for the study investigated the participants’ perceptions of online CFL learning stickiness for each latent factor through 20 items. All items are measured via a five-point Likert scale ranging from 1 (strongly disagree) to 5 (strongly agree). Considering the different native languages of all participants, who were from various countries and regions across five continents, the Chinese language was used in the online questionnaire. To ensure that all participants could correctly understand the items, the questionnaire was elaborately prepared for it could best suit participants’ current Chinese abilities. Five students with the same language level were invited before the formal investigation to answer the questionnaire. They were asked if they could fully understand the items. Then words and sentence patterns were revised in accordance with their responses. Besides, before the commencement of the online questionnaire, the researcher carefully explained the items to all participants in a tutorial class. That could minimize the misunderstanding of the questionnaire caused by participants’ language abilities and knowledge. The questionnaire included information about the procedure of the study and participants’ right to refusal. All respondents in the survey were informed that their participation was voluntary. They could withdraw from the study without reason. Their participation would not affect their final grades in the online course. Return of the questionnaire indicated students’ consent to participate.

The semi-structural individual interviews with eight students focused on their reflections, considerations, and perceptions of learning engagement and dropout in the online context. Guiding questions were set based upon the theoretical model and developed and modified by referring to student participants’ responses to the questionnaire. The interviews in the present study were organized by referring to the theoretical model and research questions, which allowed room for students to talk freely about their experiences and viewpoints of their online CFL learning.

The interviews were expected to provide some qualitative details about online learning stickiness from the eyes of students to enrich the study. Most previous studies on learning stickiness were conducted from a teacher perspective (Yang and Lin, 2020; Wang et al., 2021). As suggested by Chen (2014), investigating learning stickiness and dropout from a learner perspective is necessary as learning stickiness involves a bunch of learner and social elements related to the individual learning decisions. Incorporating interviews with students into the current study could hopefully take the learner and social dimensions into the investigation and provide a full picture of online learning stickiness. It could help answer the questions that how these latent student-related factors might affect their learning stickiness from multiple perspectives. Therefore, interviews with student participants were adopted in the present study.

### Data Collection

To address the two research questions and to enhance the quality of the mix-method research, the empirical data were gathered via an online questionnaire and individual interviews at the end of the spring semester of 2021, when participants had finished at least 1-year online CFL learning. All participants were invited to the survey on a voluntary basis. For each item, respondents were asked to circle the response that best described their level of agreement. They were told that there were no correct or wrong choices in the questionnaire, and their anonymous participation was irrelevant to their grades for the courses as well. By comparing some of the early responses and the late ones of the survey, potential non-response bias was eliminated, as no statistical significance was found in the chi-square values (*p* > 0.05). Eventually, a total of 194 valid questionnaires (97%) were returned to this study.

After the collection and initiative analysis of the raw data from the questionnaire, eight sessions of individual interviews with students were administered. Each interview lasted for around 40 min. Considering their Chinese language levels, Putonghua was used to ensure students’ free expressions, as well as the mutual understanding of the interviewees and the researcher. All student participants of the interviews had passed HSK Speaking (HSKK) Test – Intermediate Level. And of all eight interviewees, five had passed HSK Speaking (HSKK) Test – Advanced Level. That indicated that interviewees could understand the conversations, and express themselves freely in oral Chinese. Notes were taken and all interviews were tape-recorded. The transcriptions of the data were translated by professionals and double-checked with all the interviewees. A back-translation approach also enhanced its validity and reliability.

### Data Analysis

A two-step approach was adopted for the statistical analysis of the collected quantitative data: all scale variables were assessed by using Confirmatory Factor Analysis (CFA). Scale item loadings, reliability, and convergent and discriminant validity were performed for the latent variables through the CFA. Relationships among all the four major constructs and other relevant variables in paths across two time points. An oblimin solution was used as the rotation technique, as latent factors involved in this study were about participants’ perceptions of online CFL learning, which were likely correlated. The number of components to retain was determined by examining the number of eigenvalues > 1, the scree plot of eigenvalues, significant factor loadings, and the substantive interpretability of the factors (Horn, 1965). Items with low loadings or low communality were removed. Then all involved variables and the relationships within were explored by performing the Structural Equation Model (SEM) for the estimate of the theoretical model. SPSS 26.0 and AMOS 26.0 were employed for descriptive statistics and the SEM, respectively.

As for qualitative data gathered from the interviews, various methods were employed for data analysis, including coding, categorization, and triangulation. Guided by the two research questions, the participants’ descriptions in the interviews were coded based on Yang and Lin (2020)’s coding scheme, which considered the factors involved in the learning process. Four themes were adopted: (1) perceived confirmation of Chinese language learning expectancy; (2) quality and perceived usefulness of the online learning; (3) thinking on the effectiveness and efficiency of social interaction during the online learning process; and (4) experiences of technical problems and students’ preparation for the online educational technologies. In addition, to enhance the reliability of coding, a colleague of the researcher, who was also an experienced researcher in a related field, was invited to code part of the data. Results were compared. The inter-coder agreement rates for the four themes were 97.8, 96.2, 92.3, and 100%, respectively. That indicated the coding scheme worked well in the present study. After a discussion with the co-coder, the discrepancies were resolved, and the researcher coded the rest of the data. Coding of the data was informed and categorized by the theoretical model employed in the present study, which incorporated four constructs of the affecting factors of learning stickiness in an online CFL context: academic integration, social integration, expectancy confirmation, and technological factors. Data from interviews was analyzed and interpreted through content analysis. Primary findings from both qualitative data and quantitative data were triangulated. The final findings were used to address the two research questions of the study.

## Results

### Factors Affecting Online Chinese as a Foreign Language Learning Stickiness

Table 3 displays the descriptions and CFA of all scale items in the questionnaire, revealing the affecting factors of online CFL learning stickiness of international students. The loadings for all constructs were above 0.70, meanwhile, data also demonstrated that the internal consistency of the proposed model was good as the reliability values for all five constructs were above the threshold line, as suggested by Hair et al. (2006). These results collectively showed that more than half of the scaled variance had been well captured by the constructs. The statistics could reflect the picture of online CFL learning stickiness and dropout as satisfactory item reliability and good measurement properties were obtained through the scale constructs of the questionnaire.

**TABLE 3:** Descriptions, loadings, and internal reliability of scale items.

Construct	Code	Scale item	Means	SD	Loadings	Reliability
Academic integration (AI)	AI 1	Online Chinese learning is of high quality.	3.86	0.65	0.84	0.791
	AI 2	By learning Chinese online, I have access to many learning resources.	3.96	0.72	0.81	
	AI 3	Online learning improves my Chinese language skills.	3.24	0.97	0.85	
	AI 4	Learning Chinese online helps my academic development.	3.57	0.89	0.78	
Social integration (SI)	SI 1	Teachers and teaching assistants can help my online Chinese learning in time.	3.76	0.94	0.88	0.843
	SI 2	I enjoy interacting with peers in online Chinese learning.	4.18	0.58	0.71	
	SI 3	I can get assistance from a wide range of sources when learning Chinese online.	4.01	0.66	0.76	
	SI 4	Online learning provides me with a real Chinese language context.	3.53	0.89	0.78	
Technological factors (TF)	TF 1	I have access to online Chinese learning.	3.59	0.74	0.84	0.811
	TF 2	I am able to deal with technical issues by myself.	3.01	0.84	0.86	
	TF 3	I know how to adjust my devices to have a good learning experience.	2.77	0.56	0.70	
	TF 4	Online learning allows me to control over my own Chinese learning progress.	3.40	0.85	0.84	
Expectancy confirmation (EC)	EC 1	I am gratified with the efficiency of online Chinese learning.	3.69	0.69	0.87	0.847
	EC 2	I am gratified with the effectiveness of online Chinese learning.	3.78	0.77	0.81	
	EC 3	Learning Chinese online meets my learning needs.	3.36	0.58	0.78	
	EC 4	I would achieve my language learning goals through online Chinese learning activities.	3.47	0.61	0.79	
Learning stickiness (LS)	LS 1	I would learn Chinese online as often as I can.	3.66	0.60	0.74	0.768
	LS 2	I would recommend online learning for other Chinese language learners.	3.47	0.97	0.79	
	LS 3	I would continue online learning when Chinese courses are finished.	3.25	0.58	0.79	
	LS 4	I do not drop out of online Chinese courses.	3.77	0.96	0.81	

Regarding the validity of the theoretical model, the values of Average Variance Extracted (AVE) for the variance captured by each construct were evaluated. Also, Composite Reliability (CR) for the convergent and discriminant validity of the five constructs was evaluated. Table 4 displays the relationships between five constructs conceptualized in the proposed theoretical model and CR coefficients. The data showed that AVE values for all five constructs were stronger than the suggested bottom line of 0.50 (Chin, 1998), and the square root of AVE for each construct was also larger than those of the inter-construct correlations. The evaluation suggested that each construct owned a closer relationship with itself than with others, so the convergent and discriminant validity was therefore supported.

**TABLE 4:** Convergent and discriminant validity values of five constructs.

Construct	AI	SI	TF	EC	LS
AI	0.86*				
SI	0.61	0.85*			
TF	0.53	0.41	0.86*		
EC	0.74	0.66	0.41	0.82*	
LS	0.75	0.64	0.44	0.80	0.81*
AVE	0.81	0.75	0.72	0.79	0.77
CR	0.75	0.74	0.67	0.72	0.75

*The square roots of Average Variance Explained (AVE).

As suggested by Chen (2014), the examination of Common Method Variance in a self-administered questionnaire is necessary. In the present study, one major variable from each construct was selected and assessed by Harmon’s one-factor test. The result showed that the highest level of covariance explained by one factor is 28.41%. Considering the 40% threshold (Podsakoff and Organ, 1986), Common Method Bias was not a concern of this study.

### The Relationships Among the Factors Affecting Online Chinese as a Foreign Language Learning Stickiness

**FIGURE 2:**
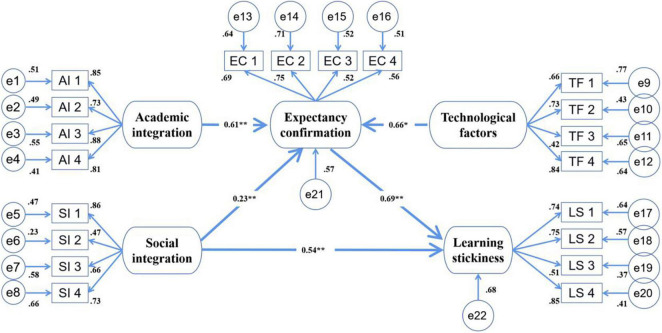
Analysis of the theoretical model. *p < 0.05 and **p < 0.001.

To explore the relationships among all the latent affecting factors of online CFL learning stickiness, an analysis of the structural model was performed. It assessed the consistency between the proposed theoretical model and those collected data. Data satisfaction was well fitted in the present study (χ*^2^*/*df* = 2.979, ^**^p < 0.001; RMSEA = 0.063; CFI = 0.94; and TLI = 0.91). CFL students’ expectancy confirmation had an important influence on online learning stickiness (β = 0.69, ^**^p < 0.001). Both academic integration and technological factors of online learning had positively correlated with students’ expectancy confirmation (β = 0.61, ^**^p < 0.001 and β = 0.66, *p < 0.05, respectively) but these two factors were found to have no significant effects on learning stickiness (β = 0.41, *p* = 0.344 and β = 0.56, *p* = 0.205, respectively). Social integration had an influential effect on learning stickiness (β = 0.54, ^**^p < 0.001), and it also had a significant correlation with expectancy confirmation (β = 0.23, ^**^p < 0.001). Moreover, expectancy confirmation and social integration in the proposed theoretical model accounted for around 68% of the variance in students’ online learning stickiness. Academic integration, social integration and technological factors together explained about 74% of the variance in expectancy confirmation. Figure 2 presents the path coefficients and explained constructs for the theoretical model in this study.

As for the qualitative data, student interviewees mentioned several factors leading to the promotion of learning stickiness or their decisions of withdrawing from the online CFL learning. As recounted by most interviewees (5/8), expectancy confirmation of international students, and their interaction with teachers and peers, were two important factors affecting their stickiness. Like most of the students, Ms. Bai, a learner of Chinese, reported that her learning stickiness was improved as she found the online learning could effectively enhance her performances in the language test:

*The online learning is full of surprises. It helps me pass HSK-5.*… *I believe the online approach is a better way for learning than the traditional in-classroom one. I failed HSK every time when I had to learn in the classroom. (Bai, Int. 3)*

Similar results were found in interviewees’ descriptions. Students would stay for learning when they found the online approach could achieve their learning expectations, including “improving their performances in HSK” (Geng, Int. 1), “solving some practical problems out of the classroom” (Jiang, Int. 5), “preparing for future job hunting” (Chen, Int. 6), and “knowing better about China and its history” (Zhang, Int. 8). Otherwise, students would “give up” (Liu, Int. 7) engaging in learning when the online learning was considered to be “useless” and “a waste of time” (Chen, Int. 6). It can be seen from the interviews that students’ engagement was closely connected with expectancy confirmation of the online CFL learning and usually reflected their learning purposes and their understanding and plans of future academic learning or career development. Online learning, as expected by many international students, was supposed to be useful for their personal needs.

Apart from expectancy confirmation, social integration of the online CFL learning was also found as an affecting factor of students’ learning stickiness. Mr. Chen was an advanced Chinese language learner, who majored in Psychology at the university, he provided his thought on social integration in the online learning:

*I preferred the online learning as it enabled me to interact with my teachers and peers 24 h 7 days.*… *In the classroom, I could only employ the class time to communicate with others. That was far from enough for a Psychology student. (Chen, Int. 6)*

Like Mr. Chen, a majority of the interviewees (6/8) listed social integration of the online CFL learning as a key factor for their preference of and engagement in learning. They described the online approach as “a time-saving way” (Qiu, Int. 2) and “a cost-effective way” (Liu, Int. 7) for interaction with teachers and peers. As language learners, they highlighted the importance of interaction using the target language, for it could “provide me with more practices” (Yang, Int. 4), and “give me opportunities to learn from others”, which could “correct my mistakes and better my pronunciation” (Geng, Int. 1). Social integration, particularly for foreign language learning, was found to be a key consideration for international students. Besides, some students (3/8) also mentioned that the online CFL learning created “a community” that gave them “a sense of belonging” (Yang, Int. 4). They enjoyed the sense and thus chose to stay for learning.

While expectancy confirmation was considered as a strong affecting factor of learning stickiness of international students in online learning, this study further found that academic and social integration were two influential factors on students’ expectancy confirmation. Mr. Geng was a Korean student who had been learning Chinese for more than 15 years. He stressed the importance of the academic quality of the online learning content from the eyes of an advanced language learner:

*The most important part, from my perspective, of a Chinese course, is not how fancy the way it presents, but how many things it can teach me. I will stay for the learning, no matter online or offline, as long as it can help achieve my learning goals. (Geng, Int. 1)*

In a similar vein, social integration could also enhance the confirmation of students’ expectancy of online CFL learning. As students said in the interviews, factors of the social integration dimension, including “making new friends” (Chen, Int. 6), “keeping good interpersonal relationships with classmates” (Yang, Int. 4), and “maintaining regular contact with teachers” (Liu, Int. 7), were some mentioned ones that could fulfill their expectations in the online classes, while “knowing better about the China society” (Qiu, Int. 2) and “practicing Chinese with native speakers” (Zhang, Int. 8) were some key reasons for their satisfaction with the online community in the learning process. For all student interviewees (8/8), social integration was believed to improve their experiences of online CFL learning.

As for the technological factors, half of the student interviewees (4/8) named them as a concern of their online CFL learning, whereas the factors were “not a major one” (Bai, Int. 3). However, for some students, technological difficulties could be still “annoying,” particularly for those who were “in a lack of necessary technical literacy” to deal with the technological problems by themselves (Qiu, Int. 2). Mr. Liu was a student with limited experience in online learning. He described his learning obstacle in the study:

*When I found the Internet connection was too unstable to support my routine learning, I was extremely anxious and disappointed. That ruined my online learning, and I did not know what I shall do.*… *I missed the old days in the classroom. (Liu, Int. 7)*

For international students like Mr. Liu, who were not fully prepared for the online CFL learning, the employment of new digital learning resources might place them into a context, where they found themselves “abandoned by the majority” (Jiang, Int. 5). The new learning would fail their expectations and bring about less satisfying learning experiences. Therefore, technological factors could be an affecting factor of students’ expectancy confirmation of the online CFL learning, and further have an indirect influence on their decisions on engagement in learning activities.

## Discussion

Encouraged by increasing affordable digital devices and ubiquitous Internet connections, technologies are and will be applied to CFL teaching and learning. To implement successful and attractive online learning, a need for identifying factors affecting international students’ engagement in online learning activities and their stickiness is proposed. As a preliminary attempt, this empirical study investigated international students’ online learning stickiness, as well as the relationships between each construct of an integrated theoretical model. The findings might contribute to current literature in the field.

### Learning Stickiness, Expectancy Confirmation, and Social Integration

As Figure 2 shows, the construct of learning stickiness signifies that expectancy confirmation and social integration had significant effects. Results indicated that international students’ expectancy confirmation of the online CFL learning was a significant factor affecting their learning stickiness. The relationships between expectancy confirmation and learning stickiness highlighted the relevance of learning expectations to their learning engagement. Once the learning could fulfill students’ learning expectations of language development and skill improvement, students would be satisfied with the outcomes and, for many of them, stay and continue their online learning with strong loyalty; that is, being sticky with online learning. This finding in a CFL context was consistent with those of some latest studies in other educational areas (Alshurideh et al., 2020).

In line with the findings from the questionnaire, students also confirmed the predicting influence of the expectancy confirmation factor on learning stickiness in the interviews. Students tended to have very high expectations of online language learning to achieve their goals, as it was usually described as an advanced tool for knowledge acquiring and skill development (Chung et al., 2020). A positive correlation could be seen between students’ Chinese learning achievement and their learning stickiness. A student would get sticky with the online learning if he/she believed the approach could effectively improve his/her Chinese language abilities in various aspects, including language tests (Geng, Int. 1; Bai, Int. 3), in-class language performance (Jiang, Int. 5), personal career development (Chen, Int. 6), and communication with the local society (Zhang, Int. 8). When students’ expectations of Chinese language learning were fulfilled, they would be largely encouraged and possibly decide to engage in the online courses more actively (Bai, Int. 3).

On contrary, a student might find the learning outcomes less satisfactory when he/she failed in confirmation of his/her preset learning expectations. Then this student would probably drop out and not return in the future. As noticed in this study, some students expected to “learn everything about the word of 打 within one class (45 min)” (Chen, Int. 6). In the Chinese language, 打 (dǎ and dá) has more than 30 meanings with different grammatical and syntactical functions and corresponding tones. It was impossible for a student to complete the task, even though abundant scaffolding materials, like pictures, videos, and audio clips, were provided in the online context. Soon these students found the learning failed their expectations and gave up engaging in learning, although the goal itself was not appropriate.

As witnessed by the present research, the story of learning 打 was not an isolated case. Students might drop out for being “disappointed” with the online learning as they might consider it “a waste of time” and thus “give up the learning” (Liu, Int. 7; Chen, Int. 6). Such thought was not fully correct but a reflection of the strong relationships between learning expectations and learning engagement. No matter what the real reasons for learning difficulties were, students might lose stickiness when their learning expectations were failed. These instances enhanced the findings from previous analysis that the learning expectancy confirmation was a key factor contributing to students’ online learning stickiness. That was also seen in other studies in education (Mills et al., 2009; Openo, 2020).

With expectancy confirmation, social integration accounted for a large part of students’ learning stickiness (*R*^2^ = 68%). Such results indicated that international students’ online Chinese learning stickiness was critically affected by social integration in addition to learning expectancy confirmation. Social integration was a key feature of online learning. Compared with the traditional in-classroom learning in China, in which a teacher-centered lecture mode is widely applied (Chen and Yu, 2019), the social integration elements of the online approach provide students with more opportunities to employ what they have learned for practical uses (Wu et al., 2011). When the online approach could possibly facilitate their social interaction in “a time-saving” and “a cost-effective” way (Qiu, Int. 2; Liu, Int. 7), students would enjoy the convenience. It was noticed that many students preferred the online Chinese courses as they could have more practice with less time and location limits (Yang, Int. 4). Incorporated with strong social integration elements, the online learning, as insisted by some student participants, could effectively improve their language skills and knowledge. This is coherent with the conclusion made in Alexander (2002), which has asserted that social integration is crucial for language knowledge building. Besides, the integration of social elements in online learning could fulfill students’ personal pursuits as well (Chen, Int. 4). From the perspective of the target language learning and personal learning expectations, students preferred the online approach and enjoyed their engagement in social interaction.

Interpersonal relationship in students’ social integration was found as another significant factor contributing to their online learning stickiness. Delahunty et al. (2014) believed that building and maintaining good interpersonal relationships was one of the fundamental concerns a student participated in online learning. Such participation was signified by “strong feelings of community” (Rovai, 2002, p.199). Driven by these feelings for social purposes, students engaged in learning actively. They answered the teacher’s questions, completed learning tasks, and submitted an assignment for “a sense of belonging” (Yang, Int. 4) – their peer students in the learning did the same. This sense of belonging, including trust between members, shared learning expectations and goals, imitations of friends, and concerns for each other, emerged as international students involved in the online community through interaction, cooperation, and negotiation with peers and teachers. For the purpose of maintaining this sense, students insisted on learning, and stickiness was elevated.

### Learning Stickiness, Academic Integration, and Social Integration

From academic integration to learning stickiness, no direct impact was found in the present study. The path was only mediated by the factor of expectancy confirmation: results from the analysis of the structural model indicated that it had a strong influence on expectancy confirmation in students’ online CFL learning (Figure 2). That indicated that students were attracted by satisfactory learning content, while they might not persist in online learning only for its high quality. Academic integration was a major concern of many students in foreign language learning. In line with previous studies (Bhattacherjee, 2001; Wang et al., 2019), students were found to be attracted by courses and resources “with high quality” that could fulfill their learning needs (Geng, Int. 1).

It should be noted in this study that, however, no statistical significance was found between the construct of academic integration and learning stickiness. Although academic integration in online learning could meet students’ expectations, and help with their language development, it did not necessarily lead to the decisions on engaging in learning or returning to learning in the future. As Garrett (2007) has concluded, “interest in online appears to be dominated by notions of convenience and is seen to imply a quality/experience tradeoff” (p. 52). Besides, for some students, academic integration attracted their engagement with learning content, instead of active participation in learning activities. As learning stickiness focuses more on the mutual relationship between learning and students, such one-side attraction is considered as “incomplete stickiness” (Robinson and Cook, 2018). This does not mean that academic integration was not important to learning stickiness, as it greatly affected expectancy confirmation, which was claimed as a strong affecting factor of learning stickiness in the current study. Academic integration had an indirect effect on learning stickiness through students’ expectancy confirmation. In this respect, the present study assumed that academic performances and learning outcomes were not the only pursuit of CFL students in their online learning. They valued their learning emotional experiences as well.

Academic and social integration were two critical latent variables for students’ expectancy confirmation. Together with technological factors, the significant influence was confirmed by sound evidence (*R*^2^ = 74%). As an affecting factor, social integration could play a role in improving experiences in the online learning context, fulfilling students’ learning expectations. The social factor in the online learning process also directly increased students’ learning stickiness. Social integration was described as one of the most critical components of successful and pleasant online learning (Abrami et al., 2011). Being involved in interaction with peers, teachers and native speakers, students are to create a supportive environment with an anxiety-free climate (Gu et al., 2021). Harmonious interpersonal relationships have been recognized as a key factor that encourages students’ learning, and resources in and outside the classroom should be combined to facilitate their Chinese language learning (Gong et al., 2021a,b). It was also noticed in this study that some students emphasized that making friends was “one of the most important tasks” of their engagement in the online learning activities (Chen, Int. 6). The convenience of interaction in online learning enabled students to achieve their social goals of keeping good relationships with peers and teachers (Yang, Int. 4; Liu, Int. 7). Moreover, interaction, as found in the present study, stimulated the exchange of ideas, the expansion of horizons, and the construction of linguistic knowledge. In line with the findings from Johnston et al. (2005) that social interaction in this environment is the key to learning effectiveness, as well as a valid predictor of a learning experience, the current study noticed the affecting role social integration played on students’ learning experiences and language knowledge construction (Qiu, Int. 2; Zhang, Int. 8). Once students believed that online learning could provide effective social integration contextualized in a relaxing environment, they would be more gratified with the approach, and the learning and practice of language knowledge. That would lead to their commitment to online learning in the future.

### Learning Stickiness and Technological Factors

As shown in Figure 2, technological factors had a significant influence on students’ online learning expectancy confirmation, which further led to their decisions on staying or withdrawing from learning activities, whereas the factor did not directly explain learning stickiness for it was not a major concern for most students (Bai, Int. 3). This finding is quite different from those of many previous ones on online learning and technology-enhanced education (e.g., Zhang et al., 2012; Chen, 2014).

Internet technology could provide students with ubiquitous access to multimedia CFL learning resources and teacher-student and student-student interaction to improve the effectiveness. Students would be satisfied with their online learning experiences when they had a stable connection to the Internet. When they had technological difficulties in engagement, however, they would find the learning fail their online learning expectations and ruined their learning experiences (Liu, Int. 7). With diverse socio-economic conditions and backgrounds, international students, particularly those who are currently learning from home, usually had different levels of access to the online community. The reliability of the Internet connection and digital devices was an essential factor affecting the effectiveness and experience of their language learning in the context. It is vital to deliver online learning in appropriate formats or means that are best suited to students’ current hardware conditions (So and Brush, 2008). Otherwise, students would find themselves being “abandoned” (Jiang, Int. 5), lose interest in the online courses, and then, in some cases, students might be reluctant to engage in learning activities anymore.

Apart from the learning equipment and Internet connection, the present research also revealed that students’ technical literacy was also an affecting factor in their online CFL learning. This finding is consistent with those in previous studies regardless of the target languages (Wu and Wang, 2005; Wu et al., 2010a). To accept the implementation of the online approach, students were expected to have sufficient technical literacy and develop an ability to apply technologies for learning purposes. The study noticed some students spent a lot of the class time dealing with software glitches and did not listen to the lectures at all. This student found the online learning could hardly meet their expectation, nor could it fulfill their learning needs, as they acquired less from it. Indeed, there were a group of international students being a lack of necessary technical literacy (Qiu, Int. 2). These students had been accustomed to traditional in-classroom instruction methods. For them, online learning was considered to be a struggle, which was different from what they had expected – online learning is supposed to be an intelligent and automated approach with many benefits. A key concern for these students was how to effectively and efficiently employ these technologies to serve their language learning and practice purposes.

Investigation noticed that most participants of the present study (84%) had an above-average level of accessibility to the Internet connection and had the necessary equipment for online learning (see Table 1). For most current online learning platforms and services, although they present in various forms with different resources, the underlying logic of learner operation is similar, like basic clicking, double-clicking, dragging, and swiping (Hoffman and Paciga, 2014). Considering the majority of these international college students were characterized as the “Generation Z” and “digital natives,” who were familiar with technologies and the operation of various kinds of devices and applications, they were supposed to be able to set an appropriate environment for the online learning with necessary assistance from teachers and computer engineers, as well as to use these online learning platforms successfully. Technological factors, both their technical literacy and the accessibility to the online community, were not a continuous concern of their engagement in the online learning. Technological factors were only found as a prerequisite of online learning. This study believes that once the technical problems are solved, factors of technological issues will not be obstacles for students’ sustainable learning and future participation in learning activities.

## Implications and Conclusion

This study explored the factors affecting international students’ online CFL learning stickiness in a Chinese university context. A developed theoretical model and the structural equation modeling analysis and individual interviews indicated that the stickiness was significantly impacted by students’ expectancy confirmation, which was closely correlated with three key factors: academic integration, social integration, and technological factors. Moreover, social integration was found to be a direct contributor to learning stickiness, whereas no significant relationships were found between academic integration and learning stickiness, and between technological factors and learning stickiness.

Theoretically, compared with some previous studies on learners in the context of general education (e.g., Halilovic and Cicic, 2013), the theoretical model of this study was designed from both learner and non-learner perspectives. Being enlightened by SCT, three dimensions and four major constructs were taken into account when investigating learning stickiness. At the same time, based on a good explanation in this study, the proposed model can be considered as a theoretical perspective to accumulate the theoretical rationales from a wide range of factors, as well as presenting a systematic picture of students’ online learning stickiness from different perspectives. In this regard, online language learning is a crucial means for learners to be integrated into an imagined community through their investment in the digital context (Stranger-Johannessen and Norton, 2017). Moreover, the above theoretical model could be used to explain CFL students’ stickiness and perceptions on the new learning and teaching approach. The effective ways, rather than some single factor, need to be systematically considered to enhance students’ engagement and persistence in learning activities. Findings from today’s Internet era and its applications in instructions may hopefully contribute to advancing the understanding of international students’ learning-related decisions and their engagement and dropout in online CFL learning in this regard.

Pedagogically, the empirical results proposed several practical implications for the purpose of providing a comprehensive insight into students’ stickiness in online CFL learning. The first implication is that the quality of learning resources and the way to present them matter to online learners. The trend of supporting CFL teaching and learning by new information and computer technologies provided seamless access to learning resources in and outside the classroom (Gong et al., 2020c,2021a). Developing qualified online learning content and delivering them in appropriate ways would effectively attract students to the online learning space, and encourage their persistence in future learning. The second implication concerns interaction in online learning. Due to time differences, technological factors, and communication obstacles, it was easy for online students to find themselves learning alone and thus drop out (Chen, 2021). Implementing some ways to encourage learning-related communicative and social interactions would be necessary as well. The interactive context could benefit language learning, as both teacher-student and peer interaction could be a way for language development, and for creating a supportive and relaxing atmosphere for language practice. The third implication is to provide technical support before and during the learning process. Through necessary technical support, students would achieve their expected outcomes with a pleasant learning experience with qualified resources, thereby facilitating their stickiness in the online learning environment.

Online language learning is not a new approach for today’s university students. They have contacted and used various kinds of digital learning resources for different learning purposes. To meet their indispensable requirements of learning, the online approach has to be “intuitive, practical, and ubiquitous” (Chen, 2014). Therefore, online learning is supposed to take students’ needs into consideration before deployment and to embed factors discussed in this study within it. Otherwise, students may find the learning fail their expectations and then drop out. Efforts are expected from all participants in the learning activities, including researchers, administrators, teachers, courseware developers, and students, to comprehend the influencing factors regarding students’ learning stickiness, and thereby to make online CFL teaching and learning benefit more international students worldwide, as well as serving their lifelong language learning purposes.

In spite of the findings from this empirical study, several limitations are also in need of attention. The findings are based upon an investigation of a limited number of international students from one university. Conclusions and implications might not be generalized in other educational contexts. Moreover, this study focused on students’ perceptions of the online approach. This might narrow the scope of the investigation, and miss some critical issues in CFL teaching and learning, such as the latent influence of different language skill learning on students’ stickiness. Future studies are recommended to provide more details.

## Data Availability Statement

The original contributions presented in the study are included in the article/supplementary material, further inquiries can be directed to the corresponding author.

## Ethics Statement

Ethical review and approval was not required for the study on human participants in accordance with the local legislation and institutional requirements. The patients/participants provided their written informed consent to participate in this study. Written informed consent was obtained from the individual(s) for the publication of any potentially identifiable images or data included in this article.

## Author Contributions

CC conceived and designed the study, collected and analyzed the data, and wrote the manuscript.

## Conflict of Interest

The author declares that the research was conducted in the absence of any commercial or financial relationships that could be construed as a potential conflict of interest.

## Publisher’s Note

All claims expressed in this article are solely those of the authors and do not necessarily represent those of their affiliated organizations, or those of the publisher, the editors and the reviewers. Any product that may be evaluated in this article, or claim that may be made by its manufacturer, is not guaranteed or endorsed by the publisher.
